# Prior cesarean section is associated with increased preeclampsia risk in a subsequent pregnancy

**DOI:** 10.1186/s12884-015-0447-x

**Published:** 2015-02-13

**Authors:** Geum Joon Cho, Log Young Kim, Kyung-Jin Min, Ye Na Sung, Soon-Cheol Hong, Min-Jeong Oh, Hong-Seog Seo, Hai-Joong Kim

**Affiliations:** Department of Obstetrics and Gynecology, Korea University Guro Hospital, Korea University College of Medicine, Seoul, Korea; The Health Insurance Review and Assessment Service of Korea, Seoul, Korea; Cardiovascular Center, Division of Cardiology, Department of Internal Medicine, Korea University Guro Hospital, Korea University College of Medicine, Seoul, Korea

**Keywords:** Cesarean section, Preeclampsia, Subsequent pregnancy

## Abstract

**Background:**

To evaluate the impact of a prior cesarean section on preeclampsia risk in a subsequent pregnancy.

**Methods:**

Study data were collected from the Korea National Health Insurance Claims Database of the Health Insurance Review and Assessment Service for 2006–2010. Patients who had their first delivery in 2006 and subsequent delivery between 2007 and 2010 in Korea were enrolled. The overall incidence of preeclampsia during the second pregnancy was estimated and to evaluate the risk of preeclampsia in the second pregnancy, a model of multivariate logistic regression analysis was performed with preeclampsia as the final outcome

**Results:**

The risk of preeclampsia in any pregnancy was 2.17%; the risk in the first pregnancy was 2.76%, and that in the second pregnancy was 1.15%. During the second pregnancy, the risk of preeclampsia was 13.30% for women who had developed preeclampsia in their first pregnancy and 0.85% for those who had not. In the entire population, prior cesarean section was associated with preeclampsia risk in their subsequent pregnancy (odds ratio [OR], 1.26; 95% confidence interval [CI], 1.13–1.41). Among women with and without preeclampsia in their first pregnancy, a prior cesarean section was associated with preeclampsia risk in their second pregnancy (OR, 1.35; 95% CI, 1.09–1.67; OR, 1.23; 95% CI, 1.08–1.40, respectively).

**Conclusions:**

Our study showed that cesarean section in a first pregnancy was associated with increased preeclampsia risk in the second pregnancy. These results provide physicians with a preeclampsia risk evaluation method for a second pregnancy that they may aid counseling in patients.

## Background

Preeclampsia is a syndrome defined by the onset of hypertension and proteinuria after 20 weeks’ gestation in previously normotensive non-proteinuric pregnant women [1]. Preeclampsia is responsible for an important proportion of fetal and maternal morbidity and mortality [2,3]. All pregnant women are at risk of preeclampsia, but no single reliable and cost-effective screening test for predicting preeclampsia has been identified to date. Therefore, specific counseling based on preeclampsia risk factors is required to assess women most at risk of developing preeclampsia. Potential risk factors associated with developing preeclampsia include a history of preeclampsia, multiple pregnancies, nulliparity, preexisting diabetes, high pre-pregnancy body mass index, advanced maternal age, an interval ≥ 10 years since a previous pregnancy, and renal disease [4].

The rate of cesarean section, a major surgery in the field of obstetrics, has been increasing [5,6]. Cesarean section is well known to be associated with a significant short-term risk of specific severe postpartum complications (eg, hemorrhage requiring hysterectomy, venous thromboembolism, and major infection) and these complication-associated re-hospitalization compared with vaginal deliveries [7,8]. Moreover, it has long-term adverse effects on mothers, such as surgical adhesions and infertility or subfertility [9]. Cesarean section also has various deleterious effects on the uterus itself, such as interference with its normal involution [10] and the creation of vascular injuries [11]. Moreover, cesarean section has been known to cause significant pathological changes around the scar tissue [12]. These uterine changes induced by cesarean section account for abnormal placentation, including placenta previa and abruption in subsequent pregnancies [13].

Because the primary pathology of preeclampsia appears to be at the maternal–fetal interface and is characterized by poor trophoblastic invasion of the uterus and subsequently altered uteroplacental blood flow [14,15], uterine changes induced by prior cesarean section may interfere with normal trophoblastic invasion and uteroplacental blood flow in subsequent pregnancies, resulting in preeclampsia. However, little is known about the effect of prior cesarean section on the occurrence of preeclampsia in subsequent pregnancies. The aim of this study was to clarity the risk factors of preeclampsia in the second pregnancy, and the effect of prior cesarean section on the preeclampsia risk in a subsequent pregnancy.

## Methods

Study data were collected from the Korea National Health Insurance (KNHI) Claims Database of the Health Insurance Review and Assessment Service (HIRA) for 2006–2010. In Korea, 97% of the population is obligated to enroll in the KNHI program. Healthcare providers are required by the health insurance policies to allow HIRA to review the medical costs incurred. The remaining 3% of the population is under the Medical Aid Program. Thus, the HIRA database contains information on all claims for approximately 50 million Koreans, and nearly all information about the volume of disease can be obtained from this centralized database with the exception of procedures that are not covered by insurance, such as cosmetic surgery. Many epidemiological analyses have been published from this database. According to the Act on the Protection of Personal Information Maintained by Public Agencies, HIRA prepares the claims data by concealing individual identities. Studies using data can, therefore, be exempt from institutional review board review [16]. The database we received included an unidentifiable code representing each individual together with age, diagnosis, and a list of prescribed procedures. The study protocol was approved by the institutional review boards of the Health Insurance Review & Assessment Service (IRB No.HIRA-1587).

International Classification of Diseases, tenth Revision (ICD-10) diagnosis and procedure codes were used to identify all women who gave birth during the study period. A first pregnancy was linked to a second pregnancy during the study period. The current study included only women who had their first delivery during 2006 and their subsequent delivery between 2007 and 2010 (n = 127,723). The diagnostic criteria for preeclampsia used in Korea are blood pressure ≥ 140/90 after 20 weeks’ gestation combined with proteinuria (≥0.3 g/24 hr or ≥ +1 on a urine dipstick; ICD-10 code O14). To identify the risk factors for preeclampsia, data of the women’s characteristics such as age, multiple pregnancies (defined as twin or higher-order gestation), delivery mode (vaginal delivery or cesarean section), and time interval between the first and second pregnancy were obtained.

The overall incidence of preeclampsia during the second pregnancy was estimated. Among those with and without preeclampsia in their first pregnancy, we estimated the proportion of women who had a second pregnancy and the incidence of preeclampsia during that pregnancy.

Student’s *t* test was used to compare continuous variables between groups, while the chi-square test was used to compare categorical variables. To evaluate the risk of preeclampsia in the second pregnancy, a model of multivariate logistic regression analysis was performed with preeclampsia as the final outcome among the entire study population. We then conducted a sub-analysis stratified by preeclampsia status in the first pregnancy. A *P* value < .05 was considered statistically significant. Statistical analyses were performed using SPSS software, version 12.0 (SPSS Inc, Chicago, IL, USA).

## Results

Among 222,137 women who had their first delivery in 2006, 6,135 women with preeclampsia were identified (preeclampsia risk in the first pregnancy, 2.76%). Among 222,137 women, 127,723 women had their second delivery between 2007 and 2010, and the proportion of women with 2 pregnancies was 50.4% and 57.7% of women with and without preeclampsia in their first pregnancy, respectively. A total of 1,473 women with preeclampsia were identified (preeclampsia risk in the second pregnancy, 1.15%). The preeclampsia risk in any pregnancy was 2.17%. During the second pregnancy, the preeclampsia risk was 13.30% for women who developed preeclampsia in their first pregnancy and 0.85% for those who did not.

Compared to women without preeclampsia in the second pregnancy, women with preeclampsia in the second pregnancy had higher rates of prior preeclampsia in the first pregnancy, older age, multiple pregnancies, and prior cesarean section (Table 1). Women with preeclampsia in the second pregnancy were older and had a significantly longer interval between their two pregnancies (Table 1).

**Table 1:** **Basic characteristics of the study population**

	**Preeclampsia in the second pregnancy**		
	**No (n = 126,250)**	**Yes (n = 1,473)**	***P*** **value**
Preeclampsia in the first pregnancy (%)	2679 (2.12)	411 (27.90)	<.01
Age in the second pregnancy (years)	30.6 ± 3.4	31.34 ± 3.7	<.01
Old age (≥35 years) in the second pregnancy (%)	14 670 (11.62)	281 (19.08)	<.01
Multiple pregnancies in the first pregnancy (%)	464 (0.37)	8 (0.54)	.27
Multiple pregnancies in the second pregnancy (%)	871 (0.69)	40 (2.72)	<.01
Prior cesarean section (%)	38 419 (30.43)	623 (42.29)	<.01
Interval between the 2 pregnancies (years)	2.50 ± 0.92	2.65 ± 1.00	<.01

Data are presented as mean ± SD or N (%).

**Figure 1:**
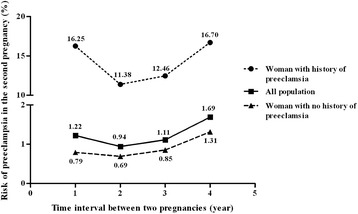
**Risk of preeclampsia according to the time interval between the two pregnancies.**

In the entire population, the risk of preeclampsia in the second pregnancy was found to decrease when there were 2-year intervals between the two pregnancies compared with 1-year intervals and then increase steadily as the time since the first delivery increased (Figure 1). According to preeclampsia status in the first pregnancy, the patterns of its risk were similar (Figure 1).

Table 2 shows the risk of preeclampsia in the second pregnancy in the entire study population. Compared to women without preeclampsia in their first pregnancy, women with a first pregnancy complicated by preeclampsia were at significantly increased risk (OR, 16.90; 95% CI, 14.93–19.12) of preeclampsia during their second pregnancy. Prior cesarean section was associated with an increased risk of having preeclampsia in the second pregnancy (OR, 1.26; 95% CI, 1.13–1.41). Old age and multiple pregnancies in the second pregnancy, and 4-year time interval between the two pregnancies were associated with an increased risk of preeclampsia in the second pregnancy, whereas a 2-year interval was associated with a decreased risk of preeclampsia compared with a 1-year interval.

**Table 2:** **Multivariate logistic regression analysis of preeclampsia in the second pregnancy**

	**Adjusted OR** ^*^	**95% CI**
Old age (≥35 years)	1.67	1.31–2.11
Preeclampsia in the first pregnancy	16.90	14.93–19.12
Multiple pregnancies in the first pregnancy	0.56	0.27–1.15
Multiple pregnancies in the second pregnancy	3.98	2.84–5.56
Prior cesarean section	1.26	1.13–1.41
Interval between the 2 pregnancies		
1 year	1	
2 years	0.80	0.68–0.95
3 years	0.97	0.81–1.15
4 years	1.48	1.24–1.77

^*^The model is adjusted for variables in the table.

OR, odds ratio; CI, confidence interval.

Table 3 shows the recurrence risk of preeclampsia in the second pregnancy of women with preeclampsia in their first pregnancy. Multivariate logistic regression analysis revealed that the presence of multiple pregnancies in the first pregnancy was associated with a decreased risk of preeclampsia in the second pregnancy. Otherwise, prior cesarean section was associated with an increased risk of preeclampsia in the second pregnancy (OR, 1.35; 95% CI, 1.09–1.67). However, old age and multiple pregnancies in the second pregnancy were not associated with recurrence risk. The time interval between the two pregnancies was significantly associated with preeclampsia recurrence risk, and the 2-year interval carried a decreased risk compared to the 1-year interval.

**Table 3:** **Multivariate logistic regression analysis for preeclampsia in the second pregnancy among women with preeclampsia in their first pregnancy**

	**Adjusted OR** ^*^	**95% CI**
Old age (≥35 years)	1.30	0.83–2.02
Multiple pregnancies in the first pregnancy	0.30	0.09–0.97
Multiple pregnancies in the second pregnancy	0.77	0.23–**2**.57
Prior cesarean section	1.35	1.09–1.67
Interval between the 2 pregnancies		
1 year	1	
2 years	0.66	0.48–0.90
3 years	0.73	0.52–1.01
4 years	1.05	0.75–1.48

^*^The model is adjusted for variables in the table.

OR, odds ratio; CI, confidence interval.

Table 4 shows the risk of preeclampsia in the second pregnancy among women without preeclampsia in their first pregnancy. Multivariate analysis of women without previous preeclampsia revealed that prior cesarean section was associated with an increased risk of preeclampsia (OR, 1.35; 95% CI, 1.09–1.67). Old age, multiple pregnancies in the second pregnancy, and 4-year interval compared with 1-year interval were also associated with an increased risk of preeclampsia.

**Table 4:** **Multivariate logistic regression analysis for preeclampsia in the second pregnancy among women without preeclampsia in their first pregnancy**

	**Adjusted OR** ^*^	**95% CI**
Old age (≥35 years)	1.88	1.43–2.48
Multiple pregnancies in the first pregnancy	1.19	0.49–2.90
Multiple pregnancies in the second pregnancy	5.01	3.58–7.01
Prior cesarean section	1.23	1.08–1.40
Interval between the 2 pregnancies		
1 year	1	
2 years	0.87	0.71–1.07
3 years	1.08	0.87–1.33
4 years	1.67	1.35–2.07

^*^The model is adjusted for variables in the table.

OR, odds ratio; CI, confidence interval

## Discussion

In this study, we found that regardless of the preeclampsia status of the first pregnancy, prior cesarean section was associated with an increased preeclampsia risk in the second pregnancy. Although the mechanism underlying this association is unclear, in our view, the most likely explanation is that the surgical cesarean section procedure increases the development of preeclampsia in subsequent pregnancy. Cesarean section has various deleterious effects on the uterus itself compared with vaginal delivery. Involution of the uterus after cesarean section is delayed compared with that after vaginal delivery [10]. Cesarean section frequently contributes to the development of post-cesarean adhesions with the bladder or pelvic wall, leading to distorted anatomy [17]. Extended uterine incisions or additional hemostatic sutures may also contribute to uterine artery injury such as pseudoaneurysm formation [11].

Moreover, cesarean section scar tissue presents significant pathological changes, including distortion of the lower uterine segment, congested endometrium above the scar recess, moderate to marked lymphocytic infiltration, residual suture material with foreign body giant cell reactions, capillary dilatation, and endometrial fragmentation and breakdown [12] as well as the biochemical behaviors of reduced pan-transforming factor-beta 3 levels and connective tissue growth factor but a slight increase in tumor necrosis factor levels [18]. The prior cesarean section was also associated with abnormal placentation, including placenta previa and abruption in subsequent pregnancies [13].

The primary pathology of preeclampsia appears to be at the maternal–fetal interface and is characterized by poor trophoblastic invasion of the uterus and subsequently altered uteroplacental blood flow [14,15]. These abnormalities may be attributed to ischemia of the placenta, which in turn releases factors into the maternal circulation that induce the clinical manifestations of the disease [19]. Therefore, these various changes in the uterus that result from surgical procedures or manipulation of the uterus during cesarean section may interfere with normal trophoblastic invasion and altered uteroplacental blood flow in subsequent pregnancies, causing preeclampsia. Similarly, Smith GC, et al. reported that prior cesarean section was associated with unexplained stillbirth in subsequent pregnancy and suggested that this association might be manifestation of abnormal uterine blood flow caused by intentional or inadvertent ligation of major uterine vessels during prior cesarean section and abnormal placentation caused by uterine scar [20].

Otherwise, several factors (i.e., maternal obesity, diabetes mellitus, etc.) which are risk factors for preeclampsia are also risk factors for having a cesarean section. Therefore, these risk factors which were responsible for cesarean section in the first pregnancy, but not cesarean section itself are attributed to occurrence of preeclampsia in the subsequent pregnancy. Moreover, a higher recurrence risk in preeclampsia is well known to be associated with earlier gestational age at the time of delivery in a prior pregnancy complicated by preeclampsia [21]. Thus, the risk of recurrent preeclampsia may simply be due to a higher risk of cesarean section in first pregnancy when preeclampsia occurred early preterm (and was severe) than when preeclampsia in the first pregnancy occurred at term and vaginal delivery was possible although prior cesarean section continues to be a mild risk factor in women without previous preeclampsia. Therefore, further studies are needed to clarify the mechanisms by which cesarean section affects the development of preeclampsia in subsequent pregnancies.

Multiple pregnancies are a well-known risk factor of preeclampsia. In line with other studies [22,23], this study found that the presence of multiple pregnancies in the second pregnancy was associated with an increased preeclampsia risk in the second pregnancy in the entire study population and even in women without preeclampsia in their first pregnancy. Moreover, among women with preeclampsia in their first pregnancy, the presence of multiple pregnancies in the first pregnancy was associated with a lower preeclampsia recurrence rate in the second pregnancy, suggesting that preeclampsia in the presence of multiple pregnancies might be caused by the fetal multiplicity itself rather than by underlying constant maternal risk factors [23]. The lack of an association between twin pregnancy in the second pregnancy with recurrent pregnancy should be interpreted with caution because the sample sizes are small in those categories (n = 28).

It has been suggested that an extended interval between pregnancies is a major risk factor of preeclampsia [24,25]. In particular, the risk of preeclampsia in the second pregnancy seemed to increase at approximately 5 years after the first delivery [25], whereas the risk after 10 years was similar to that among nulliparous women [24], suggesting that the benefit of higher parity in terms of preeclampsia risk is only transient [24]. Moreover, Trogstad et al. [25] reported that women with an inter-pregnancy interval < 1 year were at increased risk of preeclampsia compared to women with 1–5-year intervals. Similarly, the risk of preeclampsia in the second pregnancy was found to decrease in the 2-year interval compared with the 1-year interval and then increase steadily regardless of preeclampsia status in the first pregnancy. The lowest risk of preeclampsia in the 2-year interval group was seen during the first 4 years after delivery in this study. In particular, among women with a history of preeclampsia in the first pregnancy, the risk was lowest in the 2-year interval in this study.

The duration in our study was probably too short to detect the impact (if any) of a long interval between deliveries with a maximum time interval of 4 years. However, the median interval between the first and second pregnancy was 2.9 years among women with no history of preeclampsia [24], while the interval between the first and second pregnancies was <4 years in approximately 85% of women with a history of preeclampsia [21]. Therefore, these results may be useful for counseling women who are contemplating a second pregnancy, especially those who developed preeclampsia in their first pregnancy.

In this study, the rate of preeclampsia in the subsequent pregnancies of women without preeclampsia in their first pregnancy was lower (0.85%), a finding that is similar with the results of other studies (0.8%–1.8%).[21,23,25-27]. The 13.3% overall preeclampsia recurrence risk of women who had preeclampsia in their first pregnancy is also similar to the risks reported in other large population-based studies [22,25,27,28].

Prior preeclampsia was a strong predictor of preeclampsia in the subsequent pregnancy, a finding that is in line with results from other studies [21,23,26].

Several limitations should be kept in mind when interpreting our findings. First, this study was based on insurance claim data in the KNHI Claims Database, which was designed for cost claim issues, not research. Thus, the main limitation remains the validity of the data in this database. However, KNHI data has been validated in a previous study [29]. Another limitation of our study is that we were not able to access information such as maternal obesity, gestational age at first delivery, lifestyle (including smoking habits), paternity, clinical severity of preeclampsia, and neonatal characteristics, all of which are known factors of preeclampsia occurrence or recurrence rates [21,25-27], because these data were not available in the database. Nevertheless, the strength of the present study was that it used data from a population-based registry that contained all deliveries in Korea. Therefore, our results are unlikely to have been influenced by hospital type. Moreover, although odds of occurrence or recurrence in preeclampsia did not exceed 1.5 even with significant confidence intervals indicating these associations may be due to chance, clinically relevant significances may still exist.

## Conclusion

Cesarean section was associated with an increased preeclampsia risk in the subsequent pregnancy. For clinicians and women who are deciding to have an elective cesarean section on demand or without a medical or obstetrical indication, our data provide insights into the increased risk of preeclampsia in the second pregnancy.
